# Neutrophil and neutrophil-lymphocyte ratios as predictors of clinical characteristics in patients with Lymphangioleiomyomatosis

**DOI:** 10.3389/fmed.2025.1523885

**Published:** 2025-06-17

**Authors:** Lulu Yang, Liting Huang, Yingquan Luo, Siying Ren

**Affiliations:** ^1^Department of Pulmonary and Critical Care Medicine, The Second Xiangya Hospital, Central South University, Changsha, Hunan, China; ^2^Department of General Medicine, The Second Xiangya Hospital, Central South University, Changsha, Hunan, China; ^3^Research Unit of Respiratory Disease, Central South University, Changsha, Hunan, China; ^4^Clinical Medical Research Center for Pulmonary and Critical Care Medicine in Hunan Province, Changsha, China; ^5^Diagnosis and Treatment Center of Respiratory Disease, Central South University, Changsha, Hunan, China

**Keywords:** Lymphangioleiomyoma, neutrophils, neutrophil-to-lymphocyte ratio, prognosis, rare diseases

## Abstract

**Objectives:**

Lymphangioleiomyoma (LAM) is a rare multisystemic disease with variable clinical manifestations. This study aim to evaluate the potential of neutrophils and neutrophil-to-lymphocyte ratio (NLR) in predicting treatment response and prognosis in LAM.

**Methods:**

Lymphangioleiomyoma patients hospitalized in the respiratory department from January 2013 to January 2024 were retrospectively collected. Baseline data, routine blood tests, pulmonary function, and lung computed tomography (CT) were recorded, and the NLR was calculated. Patients were divided into pneumothorax and no-pneumothorax groups based on pneumothorax occurrence. Differences between the two groups were compared, and significantly different indicators were further analyzed.

**Results:**

A total of 78 patients with LAM were included in the study, 42 of them developed pneumothorax from registration to the end of follow-up, and 36 did not develop pneumothorax. There were differences in neutrophils, eosinophils, and NLR between the two groups (*P* < 0.05). Further analysis revealed that neutrophils and NLR were negatively correlated with lung function in LAM patients (*P* < 0.05), and positively correlated with lung CT grading and pneumothorax occurrence. Sirolimus treatment reduced neutrophil and NLR values in the pneumothorax group of LAM patients in (*P* < 0.05).

**Conclusion:**

Lymphangioleiomyoma patients with higher neutrophil and NLR values have worse lung function and may be more susceptible to spontaneous pneumothorax, and sirolimus treatment reduces neutrophil and NLR values in LAM patients with pneumothorax.

## 1 Introduction

Lymphangioleiomyomatosis (LAM) is a rare multisystem disease with predominantly pulmonary involvement, occurring almost exclusively in females (1). LAM has an insidious onset and progressive dyspnea, respiratory failure, and death may occur with disease progression (2). The pathogenesis of LAM is related to mutations in the *TSC1/TSC2* genes, which lead to over-activation of the mTOR pathway, the target protein of rapamycin, and thus regulate the growth of LAM cells. The mTOR inhibitor rapamycin only stabilizes the disease, and patients need to take medication lifelong, with unsatisfactory prognosis (3, 4).

Serum vascular endothelial growth factor D (VEGF-D), a biomarker of LAM disease, correlates with disease severity and can reflect the therapeutic effect of rapamycin in LAM patients (5), but controversy remains regarding the correlation between decreased VEGF-D and improved pulmonary function in sirolimus-treated patients (5, 6), and some LAM patients have normal serum VEGF-D concentrations (7). Therefore, further studies and discovery of new reliable and easily accessible biomarkers are needed to improve LAM severity and prognosis assessment.

Neutrophils, the largest component of human peripheral circulating leukocytes (8), and CD11bLy6C^+lo^Ly6G neutrophils have been identified as major myeloid cell infiltrates in LAM tumors from TSC2-deficient uterine leiomyosarcoma animal model, and inhibition of neutrophil chemotaxis and neutrophil elastase (NE) inhibits tumor growth (9). The neutrophil-to-lymphocyte ratio (NLR), calculated from complete blood counts and differentials, is an inexpensive, widely available marker associated with disease activity and prognosis in patients with chronic inflammatory diseases and malignant tumors, with high ratios in a variety of lung diseases and independent prognostic value in multiple malignancies (10–12), and association with recurrence and death in various chronic diseases (e.g., chronic obstructive pulmonary disease, idiopathic pulmonary fibrosis). However, the correlation of neutrophils or NLR with LAM clinical characteristics and prognosis has not been evaluated, and the present study aimed to assess the value of neutrophils and NLR as markers of disease severity and prognosis in LAM.

## 2 Materials and methods

### 2.1 Research design

The study was a single-center, retrospective cohort study, and the protocol was approved by the Medical Ethics Committee of the Second Xiangya Hospital of Central South University (LYEC2024-0141), with all participating patients providing informed consent. The study population included LAM patients hospitalized in our respiratory department from January 2013 to January 2024, and all patients met the American Thoracic Society (ATS)/Japanese Respiratory Society (JRS) criteria for a definitive LAM diagnosis (13), and the LAM patients were followed up through inpatient, outpatient or telephone follow-up after discharge. Patients without baseline data, including those who had already taken sirolimus before their first registration at our hospital with no traceable pre-treatment data, those who had infections at the time of initial registration and started sirolimus treatment after their first discharge, or those with only outpatient records, were excluded.

### 2.2 Data collection

Patient demographic information (gender, age, body mass index, comorbidities, duration of sirolimus use, VEGF-D), pre-and post-treatment pulmonary function tests, 6 min walk test (6MWT), lung computed tomography (CT), and baseline and post-treatment blood markers (leukocytes, neutrophils, eosinophils, and lymphocytes) were collected. The NLR defined as the ratio of the absolute counts of peripheral blood neutrophils to lymphocytes.

The number of pneumothoraces experienced by patients from symptom onset to the last follow-up visit was recorded. Pneumothorax diagnosis was based on chest imaging, and the date of pneumothorax was collected and confirmed. Recurrence of pneumothorax within two weeks was recorded as the same event, the numerator was defined as the number of pneumothorax occurrences during the observation period, and the denominator was the sum of follow-up person-years. Patients underwent CT lung examinations and were graded strictly according to previous literature (14). Mild disease was defined by cysts involving less than one-third of the lungs (grade 1); moderate disease, cysts involving two-thirds of the lungs (grade 2); and severe disease, cysts involving more than two-thirds of the lungs (grade 3).

### 2.3 Criteria for grouping

Patients were categorized into pneumothorax and non-pneumothorax groups based on the presence of pneumothorax. Demographic, clinical, and hematological characteristics of the two groups were compared.

### 2.4 Patient follow-up

Patients with stable conditions who did not meet the sirolimus usage standard (13) were followed up once every 6 months. For patients using sirolimus, the blood concentration of sirolimus needed to be checked 2–4 weeks after medication. The initial follow-up could be conducted once every 3 months, and once stabilized, patients are advised to return for follow-ups every 6–12 months based on their individual condition. The measurement of blood markers and the follow-up time are shown in Supplementary Table 1.

### 2.5 Statistical analysis

Data were analyzed using Prism 9.0 (GraphPad Software, San Diego) and SPSS Statistics 23 (SPSS Inc, Chicago). Categorical variables were expressed as numbers and percentages and analyzed using the chi-square test. Normal distribution was assessed using the Shapiro-Wilk test. Normally distributed continuous variables were expressed as mean ± standard deviation (SD) and analyzed using the two-sample Student’s *t*-test; non-normally distributed continuous variables were expressed as median and interquartile range (IQR) and analyzed using the Mann-Whitney U test. Statistical significance was defined as *P* < 0.05.

## 3 Results

### 3.1 General characteristics of the baseline

**FIGURE 1:**
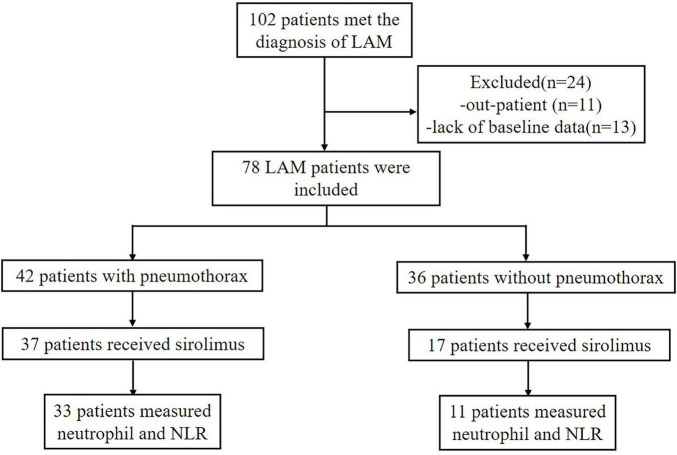
Lymphangioleiomyoma (LAM) patient enrollment results.

A total of 102 patients met the diagnosis of LAM in this study, but only 78 patients were hospitalized in our center and had more detailed baseline data recorded Figure 1. The baseline demographic data of the included patients are shown in Table 1. All patients were female and most had S-LAM. The largest number of the 78 patients had combined renal smooth muscle lipomas and retroperitoneal smooth muscle tumors (*n* = 30). Among them, patients in the pneumothorax group accounted for 70% *(n* = 21), while those in the non-pneumothorax group accounted for 30% (*n* = 9) (Table 2). Other comorbidities included rheumatic diseases (pneumothorax = 3, non-pneumothorax = 5), uterine leiomyoma (pneumothorax = 12, non-pneumothorax = 8), breast nodule (pneumothorax = 3, non-pneumothorax = 1), and hypertension (pneumothorax = 2, non-pneumothorax = 4). Eight of the 78 patients had serum VEGF-D level less than 800 pg/ml (pneumothorax = 3, non-pneumothorax = 5).

**TABLE 1:** Baseline study data.

Parameter	Total *n* = 78
Age (year)	41 ± 9.1
Body mass index (kg/m^2^) median	21.4 ± 3.25
TSC-LAM *n* (%)	9 (11.5)
Renal angiomyolipoma history *n* (%)	30 (38.5)
Chylothorax history *n* (%)	11 (14.1)
Retroperitoneal mass history *n* (%)	22 (28.2)
Serum VEGF-D (pg/ml) median (IQR)	2357 (961.5–3341)
**Pulmonary function**	
FEV1% predicted (%) median (IQR)	75 (51.7–91)
FVC% predicted (%) median (IQR)	90.6 (81.55–107.95)
DLCO% predicted (%) median (IQR)	56 (40–75.2)
6MWT distance (meter) median (IQR)	436 (360–524)
**CT grade**	
Grade I *n* (%)	28 (35.9)
Grade II *n* (%)	16 (20.5)
Grade III *n* (%)	29 (37.2)
**Blood leukocytes^a^**	
Neutrophil (× 10^9^/L) median (IQR)	4.17 (3.18–5.06)
Lymphocyte (× 10^9^/L) median (IQR)	1.64 (1.22–1.93)
NLR median (IQR)	2.32 (1.99–3.62)
Eosinophil (× 10^9^/L) median (IQR)	0.09 (0.05–0.19)
White blood cell (× 10^9^/L) median (IQR)	6.04 (5.2–7.14)

***^a^*** Baseline blood test results before the patient was not on sirolimus. Data are presented as median (lower quartile–upper quartile) or numeric (percentage). TSC-LAM, tuberous sclerosis-associated LAM; VEGF-D, vascular endothelial growth factor; FEV1, forced expiratory volume in one second; FVC, forced vital capacity; DLCO, carbon monoxide diffusion capacity; 6MWT, 6 min walk test; NLR, neutrophil-to-lymphocyte ratio.

**TABLE 2:** Baseline characteristics of patients with Lymphangioleiomyoma (LAM).

Parameter	With pneumothorax (*N* = 42)	Without pneumothorax (*N* = 36)	*P-*value
Age at presentation (year) median	32.4 ± 8.79	40.51 ± 10.73	< 0.01
Age at diagnosis (year) median	36.7 ± 8.66	41 ± 9.49	0.037
Body mass index (kg/m^2^) median	21.0 ± 3.56	21.78 ± 2.99	0.33
TSC-LAM *n* (%)	8 (15.1)	1 (4)	–
Renal angiomyolipoma history *n* (%)	21 (39.6)	9 (36)	0.7589
Chylothorax history *n* (%)	6 (11.3)	5 (20)	0.3040
Retroperitoneal mass history *n* (%)	13 (24.5)	9 (36)	0.2934
Serum VEGF-D (pg/ml) median (IQR)	1831 (937–3051)	2576 (857–3341)	0.8578
**Pulmonary function**	***N* = (27)**	***N* = (32)**	
FEV1% predicted (%) median (IQR)	60.66 (40.8–74.3)	85.01 (71.67–97)	<0.01
FVC% predicted (%) median (IQR)	83 (68.9–89.3)	100.3 (87.75–113.49)	<0.01
DLCO% predicted (%) median (IQR)	47 (33–61.4)	67(43–81)	0.018
6MWT distance (meter) median (IQR)	418 (357–514)	443 (337.25–528.5)	0.9488
CT grade			0.022
Grade I *n* (%)	11 (26.19)	19 (44.4)	–
Grade II *n* (%)	12 (28.57)	7 (19.4)	–
Grade III *n* (%)	19 (45.24)	10 (36.1)	–
**Blood leukocytes^a^**			
Neutrophil (× 10^9^/L) median (IQR)	5.15 (4.37–6.56)	3.83 (3.18–4.5)	0.0398
Lymphocyte (× 10^9^/L) median (IQR)	1.94 (1.42–1.48)	1.29 (0.17–2.05)	0.474
NLR median (IQR)	2.53 (2.30–4.87)	2.14 (1.64–2.39)	<0.01
Eosinophil (× 10^9^/L) median (IQR)	0.13 (0.07–0.28)	0.08 (0.04–0.16)	0.019
White blood cell (× 10^9^/L) median (IQR)	6.65 (5.26–8)	6 (5.22–7.11)	0.185

***^a^***Baseline blood test results before the patient was not on sirolimus. Data are presented as median (lower quartile–upper quartile) or numeric (percentage). TSC-LAM, tuberous sclerosis-associated LAM; VEGF-D, vascular endothelial growth factor; FEV1, forced expiratory volume in one second; FVC, forced vital capacity; DLCO, carbon monoxide diffusion capacity; 6MWT, 6 min walk test; NLR, neutrophil-to-lymphocyte ratio.

**FIGURE 2:**
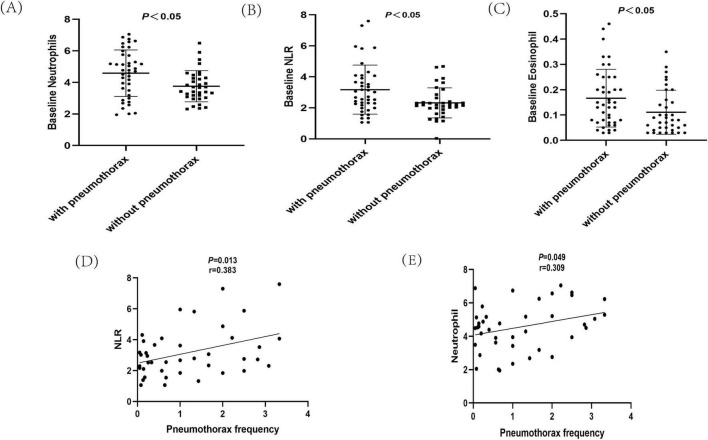
Analysis of hematological indicators in Lymphangioleiomyoma (LAM) patients. **(A)** Baseline neutrophil analysis of LAM patients with pneumothorax and non-pneumothorax. **(B)** Baseline neutrophil-to-lymphocyte ratio (NLR) analysis of LAM patients with pneumothorax and non-pneumothorax. **(C)** Baseline eosinophil analysis of LAM patients with pneumothorax and non-pneumothorax. **(D)** Analysis of NLR and pneumothorax occurrence. **(E)** Analysis of neutrophils and pneumothorax occurrence.

Repeated spontaneous pneumothorax is a major feature of LAM patients. Patients were divided into those with and without pneumothorax based on the presence or absence of pneumothorax. Among the pneumothorax group, 69.1% of patients presented with pneumothorax as their initial symptom. For the remaining patients, the median interval from their first medical visit to the first occurrence of pneumothorax was 4.8 years. Pneumothorax occurred at least twice in 47.6% of patients, and bilateral pneumothorax was observed in 42.9% of patients. The group with pneumothorax had a lower mean age of onset (32.4 vs. 40.5 years, *P* < 0.01) and mean age at diagnosis (36.7 vs. 41 years, *P* < 0.05) than the group without pneumothorax, and had a higher CT grade of the lungs and worse lung function (*P* < 0.05). The group with pneumothorax had lower pulmonary function than the group without pneumothorax, while there were no statistically significant differences between the two groups in serum VEGF-D levels and the 6 min walk test. Furthermore, the pneumothorax group had a 35.7% lung function deficit rate, and the two groups differed in baseline neutrophils, eosinophils, and NLR (*P* < 0.05) Figures 2A–C and Table 2.

### 3.2 Clinical characteristics of baseline neutrophils, eosinophils and NLR in LAM patients

The above analysis revealed differences in baseline neutrophils, eosinophils, and NLR between the pneumothorax and non-pneumothorax groups. Pearson correlation analysis showed that baseline neutrophils and NLR were negatively correlated with lung function in LAM patients, correlated with lung CT grading, but not statistically correlated with VEGF-D or 6MWT. Baseline eosinophils were not statistically correlated with lung function, CT grading, VEGF-D levels, or 6MWT Table 3. Subgroup analysis revealed that baseline NLR was negatively correlated with lung function in S-LAM patients, but the correlation did not reach statistical significance in TSC-LAM patients Supplementary Table 2, possibly due to the small number of TSC-LAM patients in this cohort. Further analysis revealed that baseline NLR (Figure 2D) and neutrophils (Figure 2E) were positively correlated with pneumothorax occurrence in patients prior to sirolimus treatment.

**TABLE 3:** Association of baseline neutrophils, neutrophil-to-lymphocyte ratio (NLR) and eosinophil with the clinical parameters in Lymphangioleiomyoma (LAM).

Parameters	Neutrophil		NLR		Eosinophil	
	R^2^	*P*	R^2^	*P*	R^2^	*P*
FEV1%	0.37	< 0.01	0.295	< 0.01	0.042	0.117
FVC%	0.34	< 0.01	0.304	< 0.01	0.068	0.058
DLCO	0.43	< 0.01	0.266	< 0.01	0.057	0.092
VEGF-D	0.05	0.146	0.005	0.63	0.012	0.57
6MWT distance	0.02	0.30	0.01	0.73	0.007	0.997
CT grade	–	< 0.01	–	0.04	–	0.329

### 3.3 Neutrophil and NLR changes in LAM patients after sirolimus treatment

**FIGURE 3:**
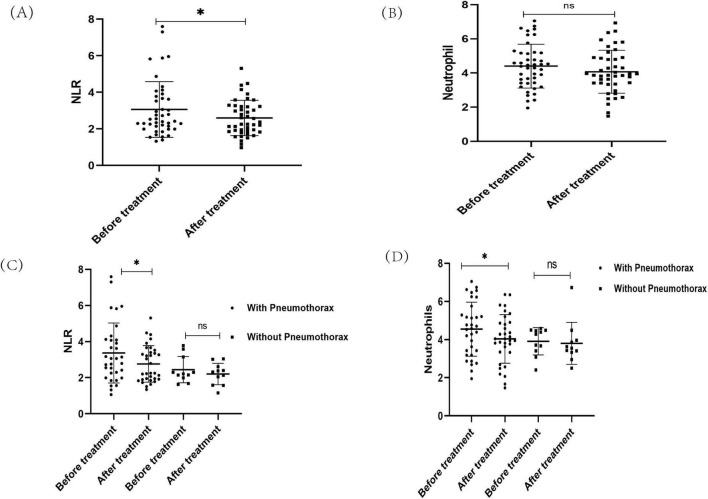
The changes of neutrophils and neutrophil-to-lymphocyte ratio (NLR) in Lymphangioleiomyoma (LAM) patients after sirolimus treatment. **(A)** NLR changes after treatment with sirolimus. **(B)** Neutrophil changes after treatment with sirolimus. **(C)** The changes of NLR after sirolimus treatment in the pneumothorax subgroup. **(D)** The changes of neutrophils after sirolimus treatment in the pneumothorax subgroup. *P < 0.05.

Sirolimus, the only currently approved therapeutic agent for LAM patients, was administered at an initial dose of 1–2 mg/day depending on the patient’s body weight and the dose was adjusted according to blood sirolimus concentration, with a target concentration in the range of 5–10 ng/ml. The neutrophil and NLR of the patients after reaching the target blood concentration(post-treatment) were recorded and compared with baseline values. A total of 54 out of 78 patients were received sirolimus, 37 in the pneumothorax group and 17 in the non-pneumothorax group, 44 of whom (33 in pneumothorax and 11 in non-pneumothorax) had a detailed record of post-treatment blood counts, which were analyzed and showed decreased NLR (Figure 3A) and neutrophils (Figure 3B) after sirolimus treatment but the decrease in neutrophils did not reach statistical significance.

Further subgroup analysis of patients in the pneumothorax and non-pneumothorax groups revealed that both neutrophils and NLR decreased in the pneumothorax group before drug treatment compared to after drug treatment (*P* < 0.05), while no significant decrease was seen in the non-pneumothorax group (Figures 3C, D). In addition, it was found that the use of sirolimus significantly reduced the incidence of pneumothorax in LAM patients (15), and in this study, 4 of 42 pneumothorax patients treated with sirolimus had pneumothorax recurrence, detailed data are shown in Table 4. Notably, patients who did not show a reduction in neutrophil and NLR after sirolimus treatment exhibited a trend toward more frequent occurrences of pneumothorax, however, this observation is based on a limited number of cases and requires further validation in larger clinical cohorts Table 4.

**TABLE 4:** Sirolimus for neutrophil and neutrophil-to-lymphocyte ratio (NLR) change in patients with recurrent pneumothorax.

No	neutrophil		NLR		Frequency of pneumothorax^a^	
	Before treatment	After treatment	Before treatment	After treatment	Before treatment	After treatment
Case 1	6.74	5.55	5.96	3.91	0.25	0.5
Case 2	2.87	4.9	1.56	2.25	0.17	2
Case 3	5.04	5.7	2.31	3.55	0.67	1
Case 4	6.57	4.3	7.3	4.39	1	1.25

*^a^*The ratio of pneumothorax occurrences to years of follow-up.

## 4 Discussion

This study aimed to evaluate the role of changes in peripheral blood neutrophils and NLR in response to disease treatment in LAM patients and their prognostic value. In this single-center retrospective study, we observed a correlation between baseline neutrophils and NLR with lung function and pneumothorax occurrence in LAM patients. Baseline neutrophils level and NLR values decreased after sirolimus treatment, but the decrease in neutrophils did not reach statistical significance. However, the analysis of patients in the pneumothorax and non-pneumothorax groups showed that both neutrophils and NLR decreased after treatment in the pneumothorax group with a significant difference, whereas the same phenomenon was not observed in the non-pneumothorax group. These results provide direct clinical support for the role of neutrophils and NLR in the pathogenesis of LAM.

To our knowledge, there are no previous studies investigating the clinical relevance of neutrophils and NLR in LAM patients. Mechanistically, our findings can be explained by experimental evidence suggesting neutrophils and NLR are involved in the pathogenesis of LAM. Based on clinical observations and basic experiments showing LAM is an estrogen-sensitive disease (3, 16). The animal models demonstrate estrogen stimulates TSC2-deficient tumor growth by promoting neutrophil production. Moreover, estrogen-promoted lung colonization of TSC2-deficient cells is dependent on neutrophils and there is a clustering of tumor-promoting neutrophils in the lungs of LAM patients (not in control patients) (17). In addition, neutrophil elastase (NE) is predominantly expressed and secreted by neutrophils, and RNA and protein analyses of TSC2-deficient uterine myometrial tumors and xenografts indicate high expression and activity of NE promoting *in vitro* growth, migration, and invasion of tumor cells (9).

Cystic changes in the lung parenchyma are characteristic lesions of LAM lungs, and patients often develop spontaneous pneumothorax (SP) (18). More than 50% of LAM patients experience at least one SP, and the chance of recurrence is about 70% (19). In our study, 25 (32.1%) patients presented with pneumothorax as their first symptom, and 17 (21.8%) developed a pneumothorax during follow-up, similar to the proportions reported elsewhere (15, 19, 20).VEGF-D serves as a current marker for LAM diagnosis and treatment response, and it has been found that patients with lower levels of VEGF-D have a higher frequency of pneumothorax (5). In our study, no difference in baseline serum VEGF-D levels was found between the pneumothorax and non-pneumothorax group, and there was no statistical difference between the two groups in 6MWT and TSC2-associated complications, these findings consistent with previous reports (15). In addition, this study found that baseline neutrophils, NLR and eosinophils differed between non- pneumothorax and pneumothorax patients, and further analysis revealed that baseline neutrophils and NLR were associated with lung function and lung CT grading. The mechanism of cyst formation by LAM cells is incompletely understood, and may be related to increased expression and activity of matrix metalloproteinases (MMPs) (21, 22), and the degradation of elastic fibers was found in the area of smooth muscle proliferation in lung biopsy specimens from LAM patients (23). LAM cells were found to secrete MMP2 and MMP9, which were elevated in patients’ serum and expressed in lung tissues in the vicinity of cysts (24, 25). Furthermore, in the inflammatory environment of tissues, neutrophils can secrete MMP to degrade the extracellular matrix (26–28). In this study, baseline neutrophils were found to correlate with lung function and CT grading, which was hypothesized to be partially related to the secretion of MMP by neutrophils to damage lung tissues. However, the specific mechanism requires further investigation.

Several limitations should be considered when interpreting the results of this study. Firstly, this was a single-center retrospective study with a relatively small number of patients included, which is partly due to the rarity of LAM. Secondly, although supplemented by detailed patient characterization and follow-up, information about pneumothorax is based in part on patient recollection, which may have been partially confounded by bias. Finally, the lack of post-treatment follow-up data for some patients presented an obstacle to subsequent analysis.

In summary, we report that baseline neutrophil levels and NLR are associated with decreased lung function, CT grading, and the occurrence of pneumothorax in patients with LAM. Peripheral blood measurements obtained from complete blood count analysis may be useful for predicting LAM disease progression and informing future management of patients.

## Data Availability

The original contributions presented in this study are included in this article/Supplementary material, further inquiries can be directed to the corresponding authors.

## References

[1] McCarthy C Gupta N Johnson S Yu J McCormack F. Lymphangioleiomyomatosis: Pathogenesis, clinical features, diagnosis, and management. *Lancet Respir Med.* (2021) 9:1313–27. 10.1016/S2213-2600(21)00228-9 34461049

[2] Gupta N Lee H Ryu J Taveira-DaSilva A Beck G Lee J The NHLBI LAM registry: Prognostic physiologic and radiologic biomarkers emerge from a 15-year prospective longitudinal analysis. *Chest.* (2019) 155:288–96. 10.1016/j.chest.2018.06.016 29940164 PMC6635733

[3] Xu K Xu W Liu S Yu J Tian X Yang Y Lymphangioleiomyomatosis. *Semin Respir Crit Care Med.* (2020) 41:256–68. 10.1055/s-0040-1702195 32279296

[4] Taveira–DaSilva A Pacheco–Rodriguez G Moss J. The natural history of lymphangioleiomyomatosis: Markers of severity, rate of progression and prognosis. *Lymphatic Res Biol.* (2010) 8:9–19. 10.1089/lrb.2009.0024 20235883 PMC2883494

[5] Young L Lee H Inoue Y Moss J Singer L Strange C Serum VEGF-D concentration as a biomarker of lymphangioleiomyomatosis severity and treatment response: A prospective analysis of the Multicenter International Lymphangioleiomyomatosis Efficacy of Sirolimus (MILES) trial. *Lancet Respir Med.* (2013) 1:445–52. 10.1016/S2213-2600(13)70090-0 24159565 PMC3804556

[6] Taveira-DaSilva A Jones A Julien-Williams P Stylianou M Moss J. Long-Term effect of sirolimus on serum vascular endothelial growth factor D levels in patients with lymphangioleiomyomatosis. *Chest.* (2018) 153:124–32. 10.1016/j.chest.2017.05.012 28533049 PMC5812752

[7] Young L Vandyke R Gulleman P Inoue Y Brown K Schmidt L Serum vascular endothelial growth factor-D prospectively distinguishes lymphangioleiomyomatosis from other diseases. *Chest.* (2010) 138:674–81. 10.1378/chest.10-0573 20382711 PMC2940071

[8] Mayadas T Cullere X Lowell C. The multifaceted functions of neutrophils. *Annu Rev Pathol.* (2014) 9:181–218. 10.1146/annurev-pathol-020712-164023 24050624 PMC4277181

[9] Taya M de la Luz Garcia-Hernandez M Rangel-Moreno J Minor B Gibbons E Hammes S. Neutrophil elastase from myeloid cells promotes TSC2-null tumor growth. *Endocr Relat Cancer.* (2020) 27:261–74. 10.1530/ERC-19-0431 32045362 PMC7394719

[10] Wareing N Mohan V Taherian R Volkmann E Lyons M Wilhalme H Blood neutrophil count and neutrophil-to-lymphocyte ratio for prediction of disease progression and mortality in two independent systemic sclerosis cohorts. *Arthritis Care Res.* (2023) 75:648–56. 10.1002/acr.24880 35287250 PMC9470772

[11] Achaiah A Rathnapala A Pereira A Bothwell H Dwivedi K Barker R Neutrophil lymphocyte ratio as an indicator for disease progression in Idiopathic Pulmonary Fibrosis. *BMJ Open Respir Res.* (2022) 9:e001202. 10.1136/bmjresp-2022-001202 35715193 PMC9207910

[12] Huang W Huang G Zhan Q Chen J Luo W Wu L The neutrophil to lymphocyte ratio as a novel predictor of asthma and its exacerbation: A systematic review and meta-analysis. *Eur Rev Med Pharmacol Sci.* (2020) 24:11719–28. 10.26355/eurrev_202011_23819 33275241

[13] McCormack F Gupta N Finlay G Young L Taveira-DaSilva A Glasgow C Official American Thoracic Society/Japanese respiratory society clinical practice guidelines: Lymphangioleiomyomatosis diagnosis and management. *Am J Respir Crit Care Med.* (2016) 194:748–61. 10.1164/rccm.201607-1384ST 27628078 PMC5803656

[14] Avila N Dwyer A Rabel A Moss J. Sporadic Lymphangioleiomyomatosis and tuberous sclerosis complex with lymphangioleiomyomatosis: Comparison of CT features. *Radiology.* (2007) 242:277–85. 10.1148/radiol.2421051767 17105849 PMC2940246

[15] Cheng C Xu W Wang Y Zhang T Yang L Zhou W Sirolimus reduces the risk of pneumothorax recurrence in patients with lymphangioleiomyomatosis: A historical prospective self-controlled study. *Orphanet J Rare Dis.* (2022) 17:257. 10.1186/s13023-022-02418-2 35804431 PMC9264575

[16] Gibbons E Minor B Hammes S. Lymphangioleiomyomatosis: Where endocrinology, immunology and tumor biology meet. *Endocr Relat Cancer.* (2023) 30:e230102. 10.1530/ERC-23-0102 37410387 PMC10529736

[17] Minor B LeMoine D Seger C Gibbons E Koudouovoh J Taya M Estradiol augments tumor-induced neutrophil production to promote tumor cell actions in lymphangioleiomyomatosis models. *Endocrinology.* (2023) 164:bqad061. 10.1210/endocr/bqad061 37042477 PMC10164661

[18] Elia D Cassandro R Caminati A Luisi F Harari S. Lymphangioleiomyomatosis. *Presse Med.* (2023) 52:104173. 10.1016/j.lpm.2023.104173 37696446

[19] Cortinas N Liu J Kopras E Memon H Burkes R Gupta N. Impact of age, menopause, and sirolimus on spontaneous pneumothoraces in lymphangioleiomyomatosis. *Chest.* (2022) 162:1324–7. 10.1016/j.chest.2022.05.036 35660029 PMC11736300

[20] Almoosa K Ryu J Mendez J Huggins J Young L Sullivan E Management of pneumothorax in lymphangioleiomyomatosis. *Chest.* (2006) 129:1274–81. 10.1378/chest.129.5.1274 16685019

[21] Moses M Harper J Folkman J. Doxycycline treatment for lymphangioleiomyomatosis with urinary monitoring for MMPs. *N Engl J Med.* (2006) 354:2621–2. 10.1056/NEJMc053410 16775248

[22] Matsui K Takeda K Yu Z Travis W Moss J Ferrans V. Role for activation of matrix metalloproteinases in the pathogenesis of pulmonary lymphangioleiomyomatosis. *Arch Pathol Lab Med.* (2000) 124:267–75. 10.5858/2000-124-0267-RFAOMM 10656737

[23] Fukuda Y Kawamoto M Yamamoto A Ishizaki M Basset F Masugi Y. Role of elastic fiber degradation in emphysema-like lesions of pulmonary lymphangiomyomatosis. *Hum Pathol.* (1990) 21:1252–61. 10.1016/s0046-8177(06)80039-0 2249838

[24] Terraneo S Lesma E Ancona S Imeri G Palumbo G Torre O Exploring the role of matrix metalloproteinases as biomarkers in sporadic lymphangioleiomyomatosis and tuberous sclerosis complex. A pilot study. *Front Med.* (2021) 8:605909. 10.3389/fmed.2021.605909 33981713 PMC8107231

[25] Revilla-López E Ruiz de Miguel V López-Meseguer M Berastegui C Boada-Pérez M Mendoza-Valderrey A Lymphangioleiomyomatosis: Searching for potential biomarkers. *Front Med.* (2023) 10:1079317. 10.3389/fmed.2023.1079317 36817769 PMC9931739

[26] Hsu A Barrett C DeBusk M Ellson C Gautam S Talmor D Kinetics and role of plasma matrix metalloproteinase-9 expression in acute lung injury and the acute respiratory distress syndrome. *Shock.* (2015) 44:128. 10.1097/SHK.0000000000000386 26009816 PMC4830084

[27] Ventura I Vega A Chacón P Chamorro C Aroca R Gómez E Neutrophils from allergic asthmatic patients produce and release metalloproteinase-9 upon direct exposure to allergens. *Allergy.* (2014) 69:898–905. 10.1111/all.12414 24773508

[28] Gilet A Zou F Boumenir M Frippiat J Thornton S Lacolley P Aldosterone up-regulates MMP-9 and MMP-9/NGAL expression in human neutrophils through p38, ERK1/2 and PI3K pathways. *Exp Cell Res.* (2015) 331:152–63. 10.1016/j.yexcr.2014.11.004 25449697

