# Supplementary material for: Neutrophil and neutrophil-lymphocyte ratios as predictors of clinical characteristics in patients with Lymphangioleiomyomatosis
Source: Front Med (Lausanne). 2025 Jun 17;12:1523885. doi: 10.3389/fmed.2025.1523885 (PMC12209317; doi:10.3389/fmed.2025.1523885)
Supplement: Supplementary file 1 [file Table_1.docx]

| **Time point** | **Measured Parameters** |
| --- | --- |
| T0 | Hematological parameters, pulmonary function tests, etc. **(baseline)** |
| T1 | The sirolimus blood concentration was measured between 2 and 4 weeks after the initiation of therapy |
| T2 | Hematological parameters, pulmonary function tests, etc. |
| T3 | Hematological parameters, pulmonary function tests, etc.  **(post-treatment)** |

**Table S1 The measurement of blood markers and the follow-up time**

T0: At hospital admission T1: 2–4 weeks after initiation of sirolimus T2: Every 3 months during the early phase of treatment; T3: Every 6–12 months during the stable phase. For patients who did not meet the treatment criteria, follow-up was conducted every 6 months during the stable phase.

**Table S2 Association of NLR with the clinical parameters in S-LAM and TSC-LAM.**

| **Parameters** | **NLR（S-LAM）** | | **NLR（TSC-LAM）** | |
| --- | --- | --- | --- | --- |
|  | **R^2^** | ***P*** | **R^2^** | ***P*** |
| **FEV1%** | 0.26 | ＜0.01 | 0.42 | 0.11 |
| **FVC%** | 0.27 | ＜0.01 | 0.36 | 0.15 |
| **DLCO** | 0.24 | ＜0.01 | 0.39 | 0.13 |
| **VEGF-D** | 0.002 | 0.78 | 0.46 | 0.21 |
| **6MWT distance** | 0.001 | 0.83 | 0.14 | 0.35 |
